# Quantifying monochromatic and polychromatic optical blur anisotropy in the periphery of myopes and emmetropes using a radial asymmetry metric

**DOI:** 10.3389/fmed.2025.1496210

**Published:** 2025-02-10

**Authors:** Chloe Degre Kendrick, Dibyendu Pusti, Geunyoung Yoon

**Affiliations:** College of Optometry, University of Houston, Houston, TX, United States

**Keywords:** myopia, emmetropization, optical anisotropy, radial asymmetry metric, peripheral blur, chromatic aberration, higher-order aberrations, astigmatism

## Abstract

**Purpose:**

The goal of this study is to characterize peripheral blur anisotropy resulting from monochromatic and chromatic aberrations along multiple meridians of myopic and emmetropic eyes using a newly developed quantitative metric.

**Methods:**

A scanning Shack-Hartmann-based wavefront sensor was used to measure lower- and higher-order monochromatic aberrations along the horizontal and vertical meridians of 20 healthy adult subjects (10 myopes, and 10 emmetropes). Monochromatic and polychromatic blur asymmetry magnitude and orientation were quantified using a novel metric based on the optical transfer function. Published population averages of longitudinal and transverse chromatic aberration were used for polychromatic blur asymmetry calculations.

**Results:**

Blur anisotropy magnitude and orientation differed between refractive groups at several peripheral retinal locations under monochromatic and polychromatic conditions. Myopes were significantly more likely to have vertically oriented blur than emmetropes under monochromatic conditions in the temporal peripheral retina beyond 20°. These differences were minimized when chromatic aberrations were included, though the trend remained the same.

**Implications:**

A trend of more vertical optical blur in the temporal periphery of myopes strengthens the hypothesis that myopes experience different peripheral optical blur than emmetropes, though the small sample size of the current study limits generalizability of the results. A thorough account of peripheral blur across the visual field may lead to a better understanding of the cues that the peripheral visual system might rely on during processes such as accommodation, emmetropization, and myopization.

## 1 Introduction

Myopia, or optical near-sightedness, is one of the leading causes of visual impairment worldwide and is linked with severe eye comorbidities that can cause permanent blindness such as maculopathy, retinal detachment, and glaucoma (1, 2). This is especially concerning due to the steadily growing prevalence of myopia, which is estimated to affect 50% of the world population by 2050 (3). Genetic factors are a known predictor of myopia development (4, 5), however, lifestyle and environment have also been shown to play a role (6). Much work has been done to identify the environmental risk factors for myopia, such as education and time spent outdoors (7–9), although the mechanism by which axial elongation occurs is still largely unexplained. Foundational animal research has found that the emmetropization and myopization processes can be impacted by visual experience, however, the precise processes by which the eye uses visual input to regulate growth in humans are not yet well understood (10–13).

The relative peripheral hyperopia (RPH) theory is one such hypothetical mechanism originating from non-human primate research suggesting that larger amounts of RPH may trigger axial elongation even when the fovea is well-corrected for defocus due to detection of residual defocus in the periphery (14). Furthermore, RPH in myopes is increased when using traditional single-vision correction (15, 16), which is thought to be an explanation for myopia progression in children wearing correction optimized only for foveal refraction. A strong association found between RPH and myopia in humans supports this theory (17, 18). In response, several treatments have been developed with the aim of reducing RPH, which have shown varying degrees of success in slowing myopia progression (19). However, longitudinal studies have not been able to predict myopia development from peripheral refraction before onset, suggesting that relative peripheral hyperopia may be an aftereffect of axial elongation, rather than a cause of myopization (20). This has prompted an investigation into other potential visual signals, such as blur orientation, that the periphery might detect as a cue for accommodation or axial elongation (21).

While peripheral refraction (i.e., lower-order aberrations) has been the primary focus in myopia control research so far, it is noteworthy that higher-order aberrations and chromatic aberrations also play a significant role in both peripheral retinal image quality and blur perception (22–25). Peripheral optical aberrations, including asymmetric aberrations such as astigmatism and coma, significantly increase in magnitude with retinal eccentricity (22, 26). Coma alone, and astigmatism when it is combined with defocus, both produce asymmetric optical blur on the retina. Notably, the blur orientation caused by astigmatism also changes direction depending on the sign of defocus it is combined with. This asymmetric blur has been hypothesized to serve as an orientational signal that aids the visual system in defocus detection and emmetropization (21, 27–29). Zheleznyak recently reported that the directionality of peripheral blur varies between refractive error groups, indicating a potential association with the development of refractive errors such as myopia (29, 30). However, this work has investigated population averages of monochromatic aberrations in the temporal peripheral retina alone. Furthermore, myopes have more relative hyperopic defocus in the periphery (17) as a consequence of their more elongated eyes, which is hypothesized to impact the shape of blur on the retina. However, the retina does not necessarily expand uniformly with myopization (31), necessitating investigation of ocular aberrations and optical quality across multiple meridians of the eye. There have been several reports of optical quality in the periphery (17, 18, 22, 24, 26, 27, 29, 30, 32–34), however, peripheral optical blur anisotropy and orientation have not been quantified using individuals’ ocular aberrations nor has there been an assessment of blur anisotropy in the nasal, superior or inferior areas of the retina.

This study aims to bridge these gaps by evaluating peripheral blur anisotropy across multiple ocular meridians while accounting for individuals’ higher-order aberration profiles. Longitudinal and transverse chromatic aberrations (LCA, TCA) are also considered in this work due to their impact on image quality (35). While LCA is mostly constant across the retina (32) TCA varies in magnitude depending on retinal eccentricity and alters blur orientation differently along different meridians (36). Furthermore, a recent study evaluating peripheral blur anisotropy at different wavelengths found differences in blur anisotropy between population-averaged aberration profiles of myopes, emmetropes, and hyperopes in the temporal peripheral retina (30).

Previous metrics have described blur anisotropy using a ratio based on the two-dimensional modulation transfer function (MTF). Zheleznyak first described optical anisotropy as the ratio of MTFs for horizontal to vertical gratings (27). Ji et al. described peripheral blur anisotropy as the ratio of overall horizontal to vertical contrast calculated by vector analysis of each modulus of the MTF filtered by the spatial resolution limit (21). Zheleznyak recently took a similar approach, by calculating the ratio of the area under the horizontal MTF divided by the area under the vertical MTF (29). The drawback of a “horizontal to vertical” (H:V) ratio-based method is that it cannot be used to quantify diagonal aberrations. Therefore, a new metric capable of characterizing blur anisotropy across the entire retina would enhance our understanding of how peripheral optics impact peripheral retinal image quality.

The current study aims to address these topics by characterizing the magnitude and orientation of peripheral blur in myopic and emmetropic individuals, considering monochromatic and population-averaged chromatic aberrations across multiple meridians of the eye. A new metric is proposed that can be used to characterize peripheral blur anisotropy and orientation in an effort to elucidate how an individual’s lower- and higher-order aberrations may interact with chromatic aberrations to contribute to peripheral blur on the retina. A more comprehensive characterization of peripheral blur in myopic and emmetropic eyes is an important step towards understanding how peripheral optics might impact mechanisms behind emmetropization and myopization.

## 2 Materials and methods

### 2.1 Subject demographics

The left eyes of 20 healthy subjects between the ages of 19 and 35 (mean: 24.8 ± 4.1) years old were included in the study (9 females and 11 males). All participants satisfied the study’s inclusion criteria, which required having healthy eyes, with no history of ocular diseases or surgeries, and no current use of medications. Most of the participants were university students and included members of our laboratory team. Subjects were sorted into two groups of ten subjects each based on cycloplegic on-axis defocus error as measured by the Shack-Hartmann wavefront sensor (26). Myopes had a mean defocus of -4.78 ± 1.47 D and emmetropes had a mean spherical refraction of 0.06 ± 0.53 D. All procedures adhered to the ethical standards of the Declaration of Helsinki and received approval from the Institutional Review Board for human subject research at the University of Rochester in Rochester, NY, USA.

### 2.2 Wavefront measurements

Each subject underwent cycloplegia and pupil dilation with one drop each of 1% tropicamide and 2.5% phenylephrine 30 min prior to wavefront measurements. Participants were positioned with a bite bar and then instructed to fixate on the center of a Maltese cross target that was co-aligned with the optical axis of a custom-built scanning Shack-Hartmann wavefront sensor for the duration of each meridional scan measurement (26). The fixation target was viewed with a cold mirror, and a lens was inserted into the optical path to correct for subjective refractive error and to control any residual accommodation remaining after cycloplegia. This inserted lens power was not included in the wavefront sensor measurement. Subjects maintained normal central fixation for the duration of each meridional scan. Wavefront data was collected using an 850 nm laser. Measured aberrations were then converted to the equivalent magnitude at 555 nm, which is the peak of the photopic CIE luminous efficiency function (37) i.e. the wavelength that the human eye is most sensitive to. For polychromatic conditions, defocus was converted to the equivalent magnitude for individual wavelengths. Further details on the measurement device can be found in a previously published paper (26). Each meridional scan was completed within five seconds, and a pupil camera was used to monitor proper alignment between the eye and the optical axis of the device during each measurement.

The measurement ranges for ocular aberrations were as follows: horizontal meridian from -30 degrees to +30 degrees in 5 degree steps and vertical meridian from -18 degrees to +18 degrees in 6 degree steps. Negative values signify nasal and inferior retinal locations, respectively, while 0 degrees designates the fovea for all scans. Zernike aberrations and wavefronts were calculated from the acquired Shack-Hartmann spot patterns at each retinal eccentricity using a 5.5 mm diameter circular pupil. A circular pupil was used for both foveal and eccentric measurements, similar to the ‘small circle’ strategy previously described by Lundström et al. (22). A point spread function (PSF) was likewise calculated from the wavefront for each individual at each tested location.

### 2.3 Chromatic aberrations

Population averages of longitudinal and transverse chromatic aberrations (LCA, TCA) induced by dispersion of light in the visible spectrum were included in our polychromatic calculations to simulate the peripheral blur that our subjects might experience in natural lighting conditions. LCA presents as wavelength-dependent defocus blur and has been shown to be relatively constant across the retina (25, 38). On the other hand, TCA increases with retinal eccentricity and has the effect of blurring the retinal image along the meridian that it is measured along. For example, TCA will cause horizontal blur along the horizontal meridian of a diffraction-limited model eye and vertical blur along the vertical meridian. TCA variation between subjects has been attributed to dislocation of the pupil center from the visual axis and TCA has been consistently found to vary linearly with eccentricity (36, 39). Furthermore, Rynders et al. found that on average, the pupil is well-centered in the human eye (40) i.e. average TCA at fovea of a population is zero. Therefore, we assumed that there was no TCA on-axis and simply applied 0.41 arcmins of TCA for every degree of eccentricity in every direction, though some previous work has found that the location of lowest TCA may be offset from the fovea (36). LCA was calculated for wavelengths between 405 and 695 nm. For the unweighted polychromatic condition, all wavelengths were equally weighted. A weighted condition with peak focus at 555 nm, corresponding to the peak of the human spectral sensitivity function (V_λ_) (37, 41) was also included.

Monochromatic calculations included only diffraction and Zernike aberrations obtained from wavefront measurements. Finally, we have used previously published cone sampling data to limit the spatial frequencies that are included in calculating blur anisotropy and orientation for all conditions (42). The asymmetries in cone spacing along the horizontal and vertical meridians were included in our processing.

### 2.4 Radial asymmetry metric

A radial asymmetry metric (RAM) used to quantify the radial asymmetry of the optical transfer function (OTF) was developed to quantitatively characterize peripheral optical blur in terms of magnitude and orientation. Unlike previous metrics (21, 29) which restrict the assessment of blur anisotropy to the ratio between horizontal and vertical components, the new RAM separately quantifies the magnitude of radial asymmetry and the directional bias of the blur (i.e., orientation). This approach provides greater flexibility, enabling the characterization of diagonal aberrations in addition to horizontal and vertical ones, across any meridian of the eye. Furthermore, this approach takes image quality into account by calculating anisotropy directly from the shape of the two-dimensional OTF matrix.

**Figure 1:**
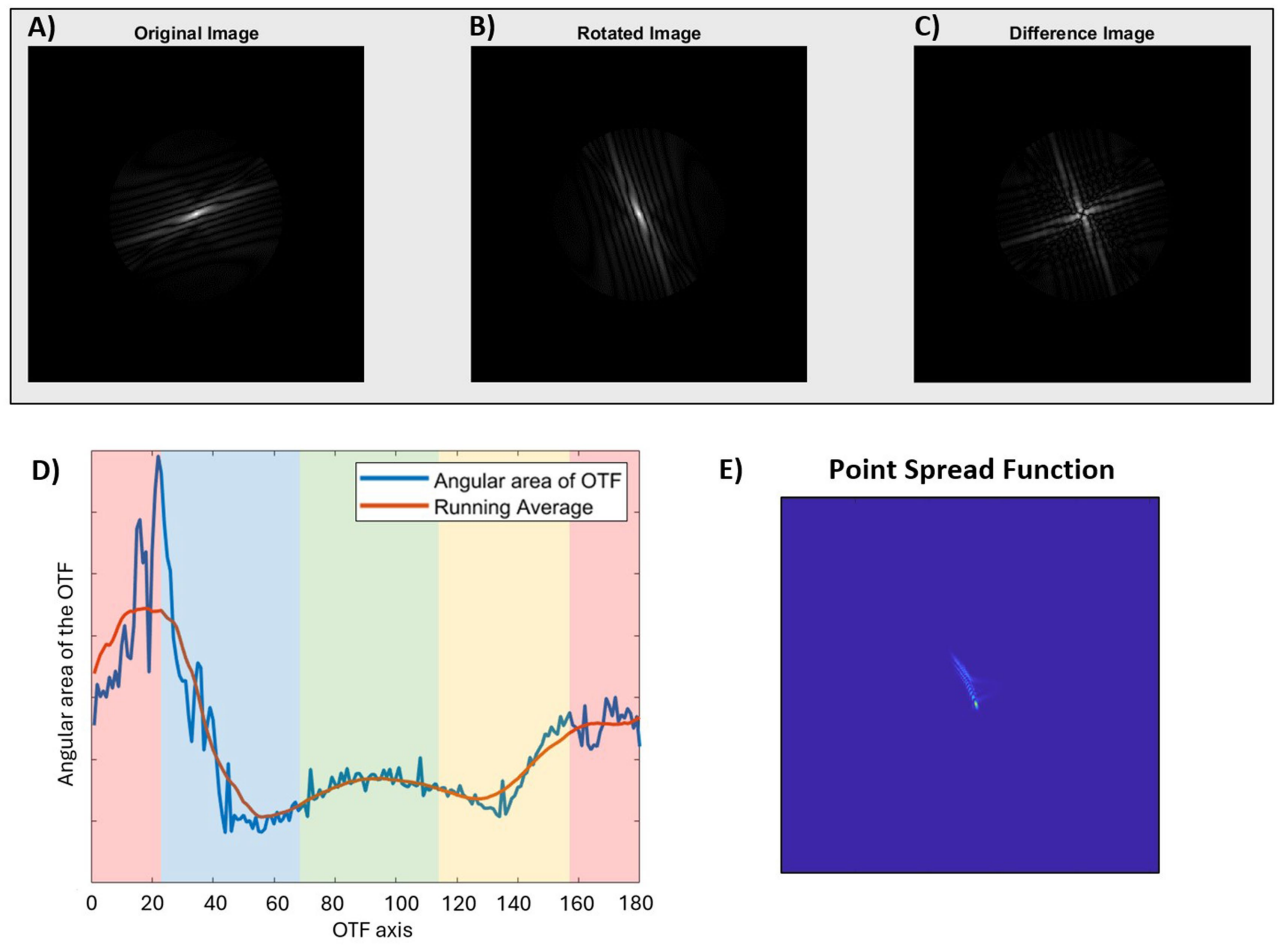
Quantification of radial asymmetry metric (RAM) magnitude **(A–C)** and orientation **(D)** of the optical transfer function (OTF), and the corresponding point spread function **(E)**. **(A)** Original OTF displaying radial asymmetry. **(B)** OTF after a 90-degree rotation to analyze asymmetry, and **(C)** Asymmetry matrix equal to the sum of the differences between the original and rotated OTF matrices. **(D)** Plot of the angular area of the OTF as a function of axis for the same OTF as shown in figure. The colored bands indicate the RAM orientation for the corresponding PSF where green is horizontal, red is vertical, blue is diagonal 135°, and yellow is diagonal 45°. **(E)** The PSF corresponding to the original image in figure. In this example, the OTF axis of maximum angular area of the OTF is 20 degrees. After accounting for the 90-degree rotation between OTF and PSF (20 + 90 = 110), the final RAM orientation is vertical (V), corresponding to a vertically blurred PSF.

RAM magnitude, i.e., the radial asymmetry of the OTF, was quantified across the horizontal and vertical meridians of the eye in five- and six-degree intervals, respectively. To do so, first, the OTF matrix was calculated from the Fourier transform of the PSF in Matlab (The MathWorks, Natick, MA). The radial asymmetry of the original OTF (Figure 1A) was then quantified by rotating the OTF by 90 degrees (Figure 1B), and then calculating the sum of the difference between the original and rotated OTF matrices (Figure 1C). It is possible to assess the radial asymmetry of the OTF in this way because of the mirror symmetry property between the first and third quadrant (and second and fourth quadrant) of the OTF matrix in the frequency domain. For example, if the original OTF was perfectly symmetric, the sum of the difference between the original and rotated OTF matrices would be equal to zero. The value was normalized by dividing the sum of the difference map (Figure 1C) by the sum of the OTF matrix of a diffraction-limited system, for that particular retinal eccentricity. In this way, the final value for RAM magnitude represents how much total asymmetry is present in the image along every direction at that specific location on the retina, with a maximum possible value of 1.

RAM orientation was also derived from the OTF by calculating the sum of the original OTF (Figure 1A) for each angular direction between 1 and 180 degrees in 1-degree angular sections. In other words, the image quality, in terms of contrast, was assessed for each axial direction of the retinal image, where a higher value indicated higher contrast or better image quality. These values were plotted along with a running average (Matlab function ‘smooth’, R2024a) to minimize the impact of noise from the matrix calculations (Figure 1D), and the axis corresponding to the maximum value from the running average was used for subsequent calculations. The axis was then converted to the retinal image perspective by subtracting (or adding) 90 degrees, so that the final reported value corresponds to the axis of blur orientation on the retina. This is analogous to the axis of maximum blur of the PSF (Figure 1E). For ease of reporting and statistical analysis, RAM orientation results were batched into one of four categories: horizontal blur (H: 1 to 22.5 and 157.5 to 180 degrees), vertical blur (V: 67.5 to 112.5 degrees), or diagonal blur (D45: 22.5 to 67.5 degrees; D135: 112.5 to 157.5 degrees).

**Figure 2:**
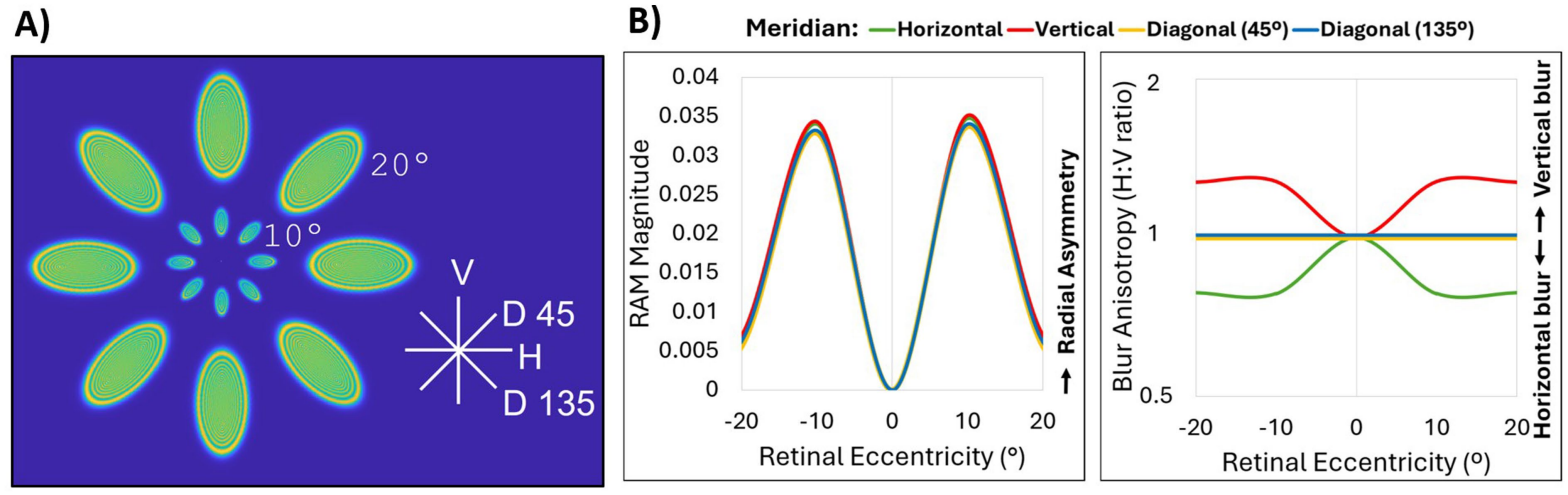
Comparison of RAM and blur anisotropy H:V ratio metrics. **(A)** Point spread functions for up to fifth-order Zernike aberrations of 0°, 10°, and 20° eccentricity obtained from a simple Zemax model eye along the horizontal (H), vertical (V), diagonal 45° (D 45), and diagonal 135° (D135) meridians. The model eye was diffraction-limited on axis with a pupil size of 4 mm and a flat retinal surface. Peripheral aberrations consisted mainly of defocus, astigmatism and coma. **(B)** Comparison of OTF-based radial asymmetry metric (RAM, left) and MTF-based H:V ratio blur anisotropy metric (right) for horizontal (green), vertical (red), diagonal 45° (yellow), and diagonal 135° (blue) meridians. All lines for RAM magnitude, and the diagonal meridian lines for blur anisotropy, overlap.

Zernike coefficients up to fifth-order aberrations were computed for central 40 degrees along the horizontal, vertical, and diagonal meridians of a simple model eye Zemax (Ansys, Canonsburg, PA). The corresponding PSFs were mapped by eccentricity and meridian (Figure 2A). RAM magnitude and blur anisotropy (Figure 2B) as described by Ji et al. (21) were computed based on the simulated on-axis and peripheral aberrations. Cone sampling limits were not included in this simulation. Figure 2 illustrates two notable differences between these two metrics. First, the RAM magnitude is the same along every meridian for the model eye, while the blur anisotropy metric does not identify anisotropy present along the diagonal meridians. This is because the blur anisotropy metric relies on quantifying anisotropy using a ratio of H:V components while the RAM quantifies overall radial asymmetry, which is the same across all meridians of a perfect model eye. Second, the blur anisotropy metric reaches a stable value beyond 10 degrees, while the RAM identifies 10 degrees as the location of peak difference with a gradual fall off towards 20 degrees. Again, the blur anisotropy calculation does not consider optical quality (or size) of the MTF. In other words, the blur anisotropy values can be the same for very different retinal image quality. Because the RAM is based on the overall size of the OTF, optical quality is accounted for in the magnitude calculation. Therefore, neither optical quality nor diagonal aberrations, which are prevalent along diagonal meridians of the model eye as shown in the corresponding PSFs (Figure 2A), can be quantified using the previous MTF-based ratio metric. Unlike the blur anisotropy metric however, RAM cannot describe magnitude and orientation in a single value. Therefore, RAM magnitude values should be interpreted alongside RAM orientation values to understand the complete description of the blur shape. For RAM magnitude shown in Figure 2B (left) RAM orientation was H along the horizontal meridian, V along the vertical meridian, D45 along the diagonal 45° meridian, and D135 along the diagonal 135° meridian.

### 2.5 Data processing and statistical analysis

Zernike analysis for each subject at each retinal location was performed using custom-built software. Lower-order aberrations (defocus and astigmatism) were corrected at the fovea for each subject to achieve a best-corrected image in terms of optical quality. The applied foveal correction was then applied to every peripheral point so that the final aberration data represented the Zernike coefficients of a well-corrected eye, similar to as if aberrations were measured with spectacle correction. This process is necessary so that peripheral blur in myopes who would typically wear refractive correction could be compared with peripheral blur in emmetropes.

JMP Pro 17 was used for all statistical analysis. A Wilcoxon nonparametric two-sample test was used to compare RAM magnitude means between refractive error groups at each eccentricity. A contingency analysis and likelihood ratio statistic following a chi-square distribution was used to compare categorical RAM blur orientation between refractive error groups at each eccentricity. A *p*-value <0.05 was considered significant and a Z-score of 1.96 was used to calculate 95% confidence intervals.

## 3 Results

### 3.1 RAM magnitude

**Figure 3:**
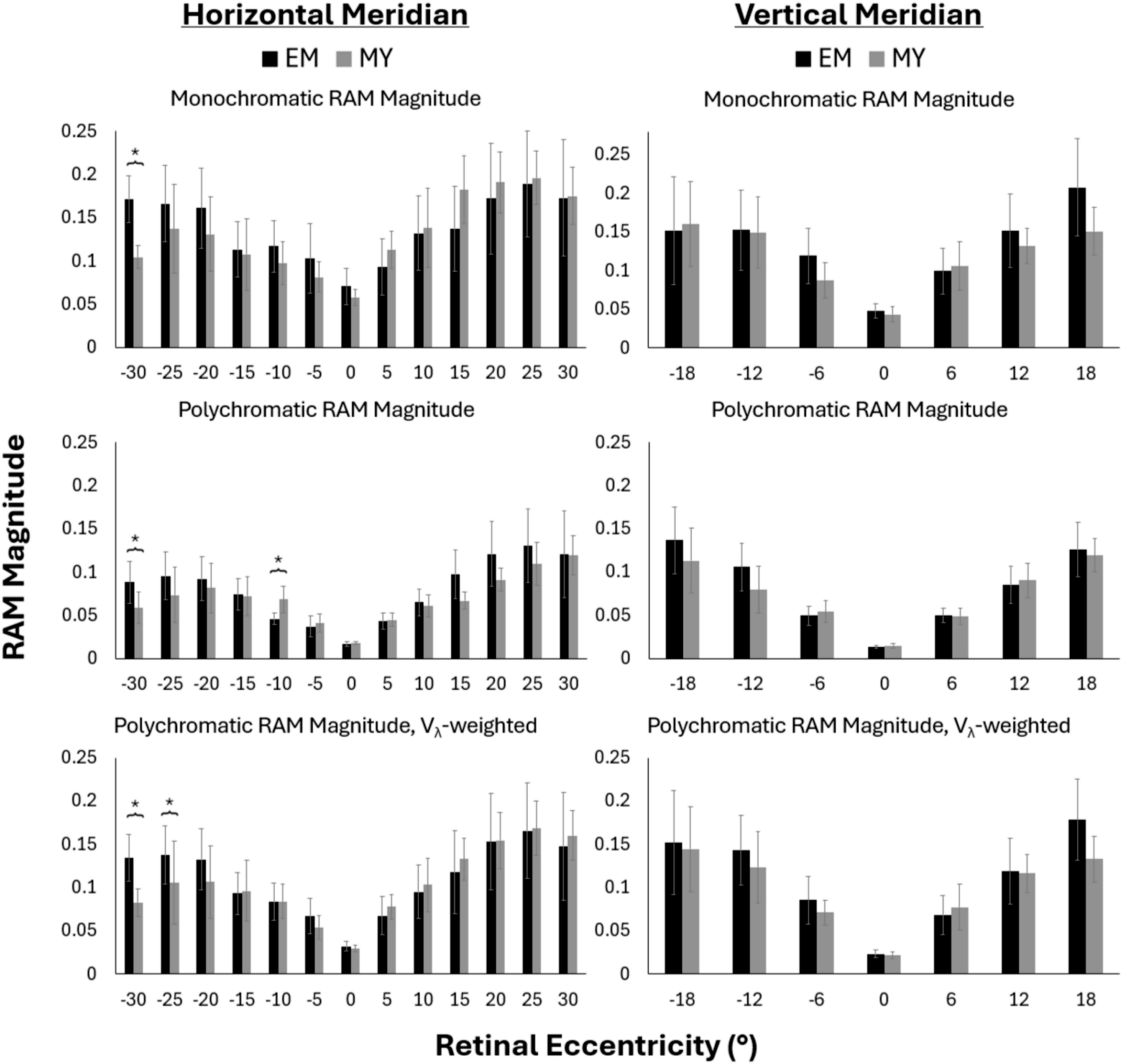
Monchromatic (top), polychromatic (middle), V_λ_-weighted polychromatic (bottom), RAM magnitude along the horizontal (left) and vertical (right) meridians for emmetropes (EM) and myopes (MY). Negative values represent nasal and inferior retinal eccentricities across the horizontal and vertical meridians, respectively. Error bars represent 95% confidence intervals. *p-value<0.05.

Three conditions were evaluated for blur anisotropy as shown in Figure 3: monochromatic (top row), polychromatic (middle row), and polychromatic weighted by the human spectral sensitivity function, V_λ_ (bottom row). Overall, RAM magnitude increased with eccentricity across both horizontal and vertical meridians. This was true for both refractive error groups across all three optical conditions. The addition of V_λ_-weighted chromatic aberrations decreased the RAM magnitude at all eccentricities. RAM magnitude was further reduced for the unweighted polychromatic condition.

Generally, the RAM magnitude was similar between myopes and emmetropes at most retinal locations (Figure 3). RAM magnitude tended to be larger in emmetropes than myopes in the nasal retina beyond 20°, though this difference was only statistically significant at nasal 30° across all three conditions (*p* < 0.05), and at nasal 25° for Vλ-weighted polychromatic. Unweighted polychromatic blur anisotropy also statistically differed between refractive groups at nasal 10° (Figure 3, middle row), the only place where myopes had significantly larger RAM magnitude than emmetropes.

### 3.2 RAM blur orientation

**Figure 4:**
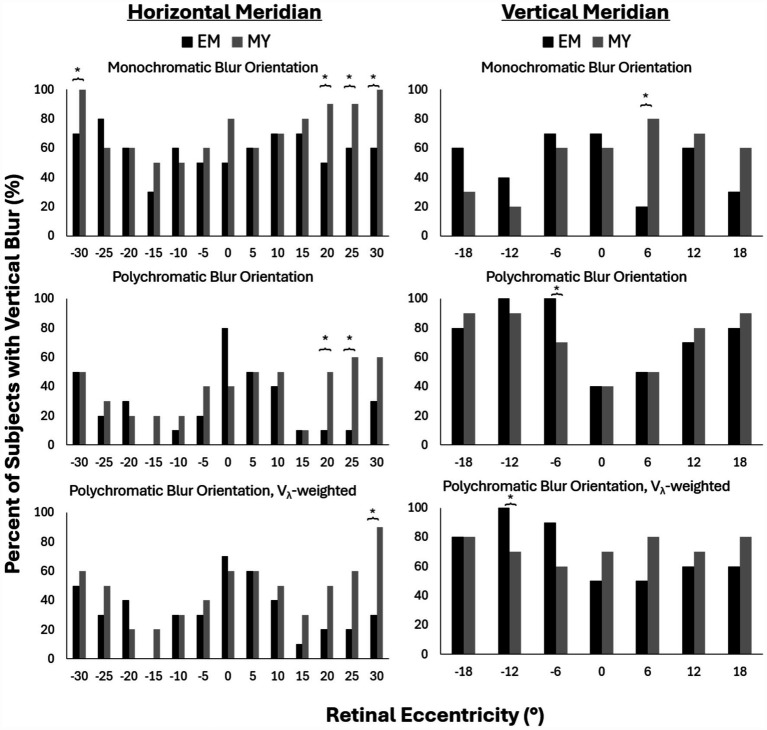
Percent of subjects with vertical blur for monochromatic (top), polychromatic (middle) and V_λ_-weighted polychromatic (bottom) conditions along the horizontal (left) and vertical (right) meridians in emmetropes (EM) and myopes (MY). Negative values represent nasal and inferior retinal eccentricities across the horizontal and vertical meridians, respectively. *p-value<0.05.

The percentage of subjects with vertical blur (as opposed to horizontal or diagonal blur) is reported in Figure 4 for the same conditions as previously described. Overall, the prevalence of vertical blur decreased with the addition of chromatic aberration in the horizontal periphery (Figure 4, left column) and increased the prevalence of vertical blur in the vertical periphery (Figure 4, right column).

Along the horizontal meridian, monochromatic conditions resulted in 100% of myopes having vertical blur at nasal 30° and temporal 30° retina compared to only 70 and 60% of myopes, respectively (Figure 4, top left). The temporal retina showed a clear trend of more myopes than emmetropes with vertical blur beyond 20°. This trend reached statistically significant differences beyond 20° for monochromatic and at 20° and 25° for the polychromatic condition (Figure 4, bottom row). V_λ_-weighted polychromatic blur orientation showed the same trend, though only reaching statistical significance at 30° temporal (Figure 4, middle row).

Along the vertical meridian, there was only one retinal location per condition that had significant differences in blur orientation between myopes and emmetropes: 6° temporal for monochromatic, 6° nasal for polychromatic and 12° nasal for V_λ_-weighted polychromatic. These differences did not appear to be part of a larger trend as the two closest rental eccentricities on either side of the locations differing between refractive groups did not exhibit similar differences between groups.

## 4 Discussion

This study quantified the magnitude and orientation of peripheral blur in myopic and emmetropic individuals, considering the effects of both monochromatic and chromatic aberrations across multiple retinal meridians, using a newly developed OTF-based metric. We confirmed that the magnitude of radial asymmetry increased with temporal, nasal, superior, and inferior eccentricity in both myopes and emmetropes. The magnitude of radial asymmetry of optical blur appeared to differ between myopes and emmetropes in the nasal peripheral retina, though a small sample size limits the statistical power of this observation. Our findings also indicate that the orientation of peripheral blur is significantly different between myopes and emmetropes in the temporal peripheral retina between 20° and 30°.

Previous studies have reported increasing blur anisotropy bias between horizontal and vertical MTF as eccentricity increases in the temporal peripheral retina (21, 29, 30). This is mostly attributed to an increase in defocus, astigmatism, and asymmetric higher-order aberrations such as coma in the periphery (18, 22, 24, 26, 34). We similarly found that optical blur in the temporal peripheral retina became more radially asymmetric as retinal eccentricity increased. We also found this to be true in the nasal, superior, and inferior retina, for all conditions. Furthermore, we observed an interesting trend that emmetropes had more radially asymmetric blur in the nasal peripheral retina than myopes, and that the difference increased with eccentricity between 20° and 30° for monochromatic and both polychromatic conditions (Figure 3, left column). Interestingly, these differences were most pronounced (reaching statistical significance at 25° and 30°) for the V_λ_-weighted condition (Figure 3, bottom left). This finding is compelling when considering peripheral blur anisotropy as a potential visual cue for emmetropization. However, this trend was not observed in the temporal retina, nor along the vertical meridian.

Recently, Zheleznyak et al. investigated chromatic cues for the sign of defocus in the peripheral retina using a large population-averaged aberration dataset and an MTF-based H:V ratio metric (30). They found that, in the temporal retina, green and red light caused vertical blur in myopes but horizontal blur in emmetropes. Our results similarly indicate that myopes have more vertical blur than emmetropes in the temporal retina when the full visual spectrum is considered. Alternatively, Zheleznyak et al. also found that blue light alone produced a horizontal blur signal in both emmetropes and myopes in the horizontal periphery. It is important to note that there are several differences between these two studies that should be considered when comparing results. Firstly, we examined the overall impact of monochromatic and chromatic aberrations from the visible spectrum on the OTF and peripheral blur rather than the impact of individual wavelengths of light. This approach provides a straightforward representation of how optical blur appears on the retina under real-life conditions, where many wavelengths of light are present simultaneously. Secondly, we used data from individuals, however, our sample sizes were small, which limits the statistical power of our study. In contrast, Zheleznyak et al. used a large population-averaged dataset, which may provide more generalizable results. Lastly, we used a 5.5 mm circular pupil for all Zernike analysis rather than a more realistic elliptical shape. A key advantage of using a circular pupil that fits into the larger ellipse created by measuring eccentric aberrations is that Zernike coefficients can be directly compared between different eccentricities (22). However, previous studies have concluded that pupil ellipticity does not significantly impact the MTF for eccentricities less than 30° (24) which is the maximum eccentricity we measured in this study. Despite the small sample size and the methodological differences between our study and previous studies, we found similar trends, which suggests that our findings are robust.

Overall, radial asymmetry decreased when chromatic aberrations were added. The effect was larger for the unweighted polychromatic condition compared to the V_λ_-weighted condition. In other words, the interaction of chromatic aberrations and monochromatic aberrations resulted in a more symmetric blur shape. One explanation is that radially symmetric LCA had more impact on the blur asymmetry than asymmetric TCA at the tested retinal eccentricities. However, this relationship might be reversed at higher eccentricities where TCA increases while LCA remains the same (36, 38). Similarly, the addition of chromatic aberrations appeared to standardize blur orientation across refractive groups, reducing the prevalence of vertical blur in the temporal periphery of myopes and increasing the prevalence of horizontal blur in both myopes and emmetropes in the horizontal periphery. Likewise, the percentage of subjects with vertical blur increased with chromatic aberrations along the vertical meridian. This was expected as TCA increases horizontal blur along the horizontal meridian and increased vertical blur along the vertical meridian. Notably, the impact of this common factor was great enough to minimize some of the statistical differences we found between groups under monochromatic conditions, though not enough to fully eliminate them. This suggests that blur orientation bias may still be markedly different between refractive error groups under natural conditions when the full visible spectrum is contributing to optical blur, especially at larger eccentricities.

We did not find any differences between refractive groups in magnitude of blur asymmetry along the vertical meridian (Figure 3, right column), and there were only standalone differences in blur orientation (Figure 4, right column). The lack of significant differences is not necessarily surprising considering that orientation only differed between refractive groups at eccentricities beyond 18° along the horizontal meridian. Aberration measurement along the vertical meridian was restricted to ±18 degrees because of the physical limitations of the scanning wavefront sensor. This is primarily due to the protrusion of the upper and lower eyelids, making it especially difficult to measure beyond 18° in the inferior retina. Alternative methods to measure aberrations or characterize the shapes of the optical surfaces of the eye beyond that range are necessary to gain a deeper understanding of optical blur in the superior and inferior periphery.

There was only one instance where RAM magnitude was found to be significantly larger in myopes than emmetropes. This was found at 10° nasal retina with unweighted chromatic aberration (Figure 3, middle left). This location is near the optic disc of the eye which is approximately at 15° nasal retina. Studies of peripheral refraction have observed localized relative myopic defocus near the optic disc of emmetropes (33). This, along with the knowledge that myopes are more likely to have a tilted optic disc (43) as well as relative peripheral hyperopia, may help to explain the significant difference found between groups at this retinal location. This finding suggests that myopes may experience different optical conditions near the optic disc compared to emmetropes, though finer sampling of monochromatic aberrations around the optic disc would be necessary to draw a conclusion.

Previous work has used MTF-based metrics to describe blur anisotropy in the periphery (21, 29), however, this strategy comes with notable limitations. While the MTF contains contrast information about the retinal image, it disregards phase information which has been shown to be important for image recognition (44), especially for broadband stimuli when higher-order aberrations are present as is the case in everyday viewing of the natural world (45). Therefore, we based our metric on the OTF which contains both contrast and phase information. Secondly, the combined effect of spectral sensitivity and decreased cone spacing in the periphery has not been included in peripheral blur studies. These conditions were included since it is not currently known if or how blur anisotropy is detected locally on the retina. We therefore used previously published cone sampling data to limit the spatial frequencies that are included in calculating blur anisotropy and orientation (42), and included a V_λ_-weighted version of the polychromatic condition (41) to simulate cone spectral sensitivity. The cone sampling limit truncated the OTF to only include lower spatial frequencies at higher retinal eccentricities, while the V_λ_-weighted polychromatic condition, a function of the spectral sensitivity of cone photoreceptors, specified how much impact each wavelength would have on the final retinal image quality metric. The function V_λ_ has a Gaussian shape with a maximum at 555 nm. Finally, H:V ratio-based metrics are limited to describing only the horizontal and vertical components of blur, which ignores the impact of aberrations that cause diagonal blur. Though not included in this work, characterization of oblique meridians is likely to yield diagonally oriented blur as shown in Figure 2A, especially when chromatic aberrations are included, due to oblique astigmatism and TCA. Therefore, the RAM described in this paper is sufficiently versatile for application to any ocular meridian of interest without bias.

Our findings also have implications for how we evaluate emerging myopia treatments. While multifocal and orthokeratology lenses are designed to decrease refractive error on the retina, they have also been found to increase higher-order aberrations on the peripheral retina (46, 47). At the same time, recent studies have claimed that contrast reduction could have a protective effect against myopia progression (48). Characterization of peripheral aberrations and consequently, blur anisotropy, with current myopia treatments may lead to a better understanding of why some optical treatments are more effective than others. Most importantly, this kind of understanding can aid the development of better and more effective myopia interventions.

Several factors were included in this analysis to reinforce the quality of the results of this study. First, individual higher-order aberrations were used to ensure that results are applicable to individual eyes. When higher-order aberrations are averaged across a large population, the individual variations tend to be minimized, leading to an underestimation of their true impact. This occurs because higher-order aberrations, other than spherical aberration, are somewhat randomly distributed within a normal population at fovea (49) and in the periphery (22). In other words, averaging these aberrations across many individuals effectively neutralizes the unique differences present in each person’s eye. These foveal individual differences are likely translated to the periphery as well, emphasizing the value of considering individualized optical treatment if peripheral optics are found to be a factor in myopia development or progression. Furthermore, some research has found that higher-order aberrations may vary between myopes and emmetropes (34). Specifically, Mathur, et al. found that the rate of change of coma increasing with eccentricity is greater in myopes than emmetropes. Notably, coma induces asymmetric blur on the retina. Secondly, multi-meridional analysis is similarly important to this investigation as imaging studies have shown that asymmetries exist in the shape of eye between different meridians (31, 32). Lastly, it is currently unknown if anisotropy or blur orientation signals are used by the retina. However, if the signal is detected and used, it may be by cone photoreceptors which have wavelength-dependent sensitivity. Therefore, chromatic aberrations, eccentricity-specific cone sampling, and the cone spectral sensitivity function were included to simulate realistic ocular optical conditions.

A limitation of our study is the small sample size, which may affect the generalizability of our findings. Additionally, while we measured aberrations along horizontal and vertical meridians, future studies should include a more comprehensive mapping of the peripheral retina, including diagonal meridians, to capture the full extent of peripheral aberrations. Population averages of chromatic aberration were used for the inclusion of both LCA and TCA in this study. Direct measurement of these factors, specifically in the periphery, may lead to more conclusive results, though some preliminary investigation by our lab has shown that the inaccuracy caused by using population-averaged chromatic aberration is minimal. A very recent study found that blur anisotropy was resistant to pupil changes for pupils larger than 1.5 mm at 30 deg. eccentricity (30). While pupil size was not a primary outcome of this study, it would be interesting for future work to assess how blur anisotropy changes with pupillary fluctuations. Finally, and most essentially, longitudinal studies are necessary to determine if there is a causal relationship between peripheral blur and refractive error.

In conclusion, our study provides valuable insights into the peripheral optical blur experienced by myopes and emmetropes under both monochromatic and polychromatic conditions. Our versatile metric can be used to precisely characterize peripheral blur orientation in any ocular meridian, which provides a useful alternative to other commonly used metrics, especially in cases where diagonal aberrations are present. The differences in peripheral blur orientation between our small groups of myopes and emmetropes underscore the importance of considering how peripheral visual signals, other than simply relative peripheral defocus, might impact myopization and emmetropization. By providing a comprehensive account of monochromatic and polychromatic peripheral blur in individual subjects, this study strengthens our knowledge of the peripheral visual signals available to the physiological systems that regulate eye growth. Future research should continue to explore these factors across different populations and under varying optical conditions to further improve our understanding and therefore lead to better myopia intervention strategies.

## Data Availability

The raw data supporting the conclusions of this article will be made available by the authors, without undue reservation.
